# Interdependence between Chromogranin-A, Alternatively Activated Macrophages, Tight Junction Proteins and the Epithelial Functions. A Human and In-Vivo/In-Vitro Descriptive Study

**DOI:** 10.3390/ijms21217976

**Published:** 2020-10-27

**Authors:** Nour Eissa, Hayam Hussein, Diane M. Tshikudi, Geoffrey N. Hendy, Charles N. Bernstein, Jean-Eric Ghia

**Affiliations:** 1 Department of Immunology, University of Manitoba, Winnipeg, MB R3E 0T5, Canada; Nour.Eissa@umanitoba.ca (N.E.); tshikudd@myumanitoba.ca (D.M.T.); 2 Children’s Hospital Research Institute of Manitoba, University of Manitoba, Winnipeg, MB R3E 3P4, Canada; 3 Section of Gastroenterology, Department of Internal Medicine, Rady Faculty of Health Sciences, University of Manitoba, Winnipeg, MB R3E 0T5, Canada; Charles.bernstein@umanitoba.ca; 4 University of Manitoba IBD Clinical and Research Centre, University of Manitoba, Winnipeg, MB R3A 1R9, Canada; 5 Department of Parasitology and Animal Diseases, Veterinary Research Division, National Research Centre, Giza 12622, Egypt; Hayam.Hussein.57@gmail.com; 6 Metabolic Disorders and Complications, McGill University Health Centre-Research Institute, Departments of Medicine, Physiology, and Human Genetics, McGill University, Montreal, QC H4A 3J1, Canada; geoffrey.hendy@mcgill.ca

**Keywords:** alternatively activated macrophages, chromogranin-A, cytokines, epithelial repair, fibrosis, gut hormones, inflammatory bowel disease, intestinal epithelial cells, macrophages, tight junction, ulcerative colitis

## Abstract

**Background:** Ulcerative colitis (UC) is characterized by altered chromogranin-A (CHGA), alternatively activated macrophages (M2) and intestinal epithelial cells (IECs). We previously demonstrated that CHGA is implicated in colitis progression by regulating the macrophages. Here, we investigated the interplay between CHGA, M2, tight junctions (TJ) and IECs in an inflammatory environment. **Methods:** Correlations between *CHGA* mRNA expression of and TJ proteins mRNA expressions of (Occludin [*OCLN*], zonula occludens-1 [*ZO1*], Claudin-1 [*CLDN1*]), epithelial associated cytokines (interleukin *[IL]-8*, *IL-18*), and collagen (*COL1A2*) were determined in human colonic mucosal biopsies isolated from active UC and healthy patients. Acute UC-like colitis (5% dextran sulphate sodium [DSS], five days) was induced in *Chga*-C57BL/6-deficient (*Chga^−/−^*) and wild type (*Chga^+/+^*) mice. *Col1a2* TJ proteins, *Il-18* mRNA expression and collagen deposition were determined in whole colonic sections. Naïve *Chga^−/−^* and *Chga^+/+^* peritoneal macrophages were isolated and exposed six hours to IL-4/IL-13 (20 ng/mL) to promote M2 and generate M2-conditioned supernatant. Caco-2 epithelial cells were cultured in the presence of *Chga^−/−^* and *Chga^+/+^* non- or M2-conditioned supernatant for 24 h then exposed to 5% DSS for 24 h, and their functional properties were assessed. **Results:** In humans, *CHGA* mRNA correlated positively with *COL1A2*, *IL-8* and *IL-18*, and negatively with TJ proteins mRNA markers. In the experimental model, the deletion of *Chga* reduced IL-18 mRNA and its release, * COL1A2* mRNA and colonic collagen deposition, and maintained colonic TJ proteins. *Chga^−/−^* M2-conditioned supernatant protected caco-2 cells from DSS and oxidative stress injuries by improving caco-2 cells functions (proliferation, viability, wound healing) and by decreasing the release of IL-8 and IL-18 and by maintaining the levels of TJ proteins, and when compared with *Chga^+/+^* M2-conditioned supernatant. **Conclusions:** CHGA contributes to the development of intestinal inflammation through the regulation of M2 and epithelial cells. Targeting CHGA may lead to novel biomarkers and therapeutic strategies in UC.

## 1. Introduction

In a homeostatic state, the colonic epithelial barrier is composed of a monolayer of epithelial cells sealed by tight junction (TJ) proteins. Its physiological role relies on regulating the passage of nutrients, water, ions, macromolecules and various antigens [1]. Disruption of the intestinal epithelial barrier significantly contributes to the pathophysiology of inflammatory bowel disease (IBD). IBD encompasses a spectrum of complex inflammatory disorders, including ulcerative colitis (UC) and Crohn’s disease (CD). They are believed to result from dysregulated innate and adaptive immune responses to gut microbes in a genetically susceptible host [2,3]. UC is a confined inflammation of the colonic mucosa affecting the rectum and variable lengths of colon, including possibly the entire colon. It is characterized by an aberrant immune response [4,5]. In UC, the intestinal epithelial barrier is characterized by increased permeability leading to the translocation of luminal microbes to the host, and a defect in mucosal repair and epithelial cell proliferation, thereby perpetuating the dysregulated immune responses [6].

The innate immune system, particularly macrophages, is critical in regulating gastrointestinal inflammation and gut permeability [2,6]. Macrophages are considered as the gatekeepers of immune homeostasis in the gastrointestinal tract. Macrophages can be divided into two major subsets: the pro-inflammatory (M1) and the anti-inflammatory (M2) cells [7]. They discriminate between harmless antigens and potential pathogens to maintain gut homeostasis [7]. M2 produce anti-inflammatory molecules (IL-10, Arginase) and subsequently play a significant role in suppressing inflammation [8]. Moreover, upon disrupting the intestinal epithelium, M2′s presence is also fundamental to re-establish the epithelial barrier by supporting epithelial cell proliferation via M2 mediators [8]. This evidence suggests a causal link between alerted monocyte-macrophage differentiation and the defection of intestinal inflammation resolution in patients with UC and CD [7]. Therefore, M2 have been considered a novel potential target for developing new treatment tactics [9,10].

Chromogranin-A (CHGA) is widely expressed by neuroendocrine cells but is also secreted by enterochromaffin cells in the digestive system [11]. CHGA is involved in regulating several biological processes within the endocrine, neuroendocrine, cardiovascular and immune systems [11]. In UC, the alteration of the intestinal epithelial homeostasis is associated with intestinal cell differentiation, including alteration in the levels of CHGA [12]. Hyperplasia of enterochromaffin cells and elevated levels of CHGA have been reported in UC and CD patients and animal models of colitis [12,13,14,15], demonstrating that CHGA plays a potentially important role in regulating intestinal inflammation through various targeted mechanisms [15,16,17]. We recently reported that chromofungin and catestatin, two CHGA-derived peptides, protect against intestinal inflammation by maintaining TJ proteins’ epithelial barrier functions and regulating M2 [6,17]. Although CHGA is linked to the pathophysiology of gut inflammation and other diseases [2,6,9,12,15,17,18,19], there are no available data describing the impact of CHGA on M2 and on the markers of the epithelial cells in the context of inflammation.

Herein, for the first time, we describe the relationship between CHGA and functional epithelial markers in human colonic mucosal biopsies isolated from UC patients. We further described the effects of the lack of CHGA on functional epithelial markers in a UC-like dextran sulphate sodium (DSS) model of colitis. Finally, we described its impact of the M2-conditioned supernatant on human colonic cell line functions.

## 2. Results

### 2.1. CHGA mRNA Expression Correlates Positively with Epithelial-Associated Cytokines & Collagen mRNA Expression and Negatively with TJ Proteins mRNA Expression Markers in UC Patients and Healthy Individuals

**Figure 1:**
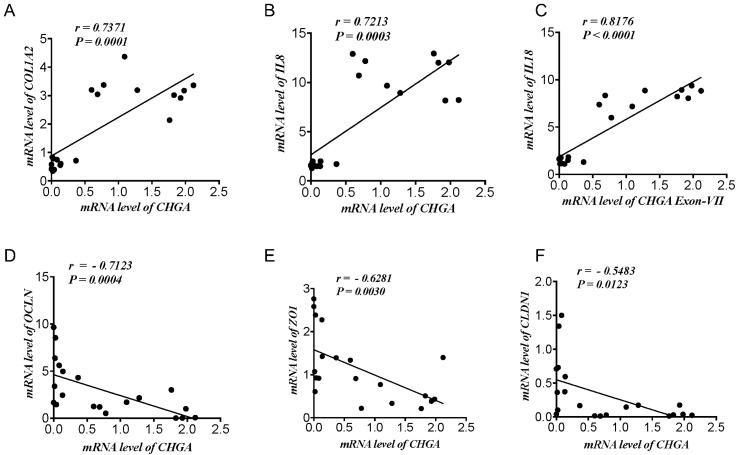
Chromogranin A (CHGA) mRNA expression correlates positively with epithelial-associated cytokines & collagen and negatively with tight junction (TJ) proteins in ulcerative colitis patients and healthy individuals. Correlation analysis between *CHGA* mRNA levels expression and (**A**) collagen (*COL12A*) (**B**,**C**) epithelial associated cytokines (*interleukin [IL]-8*, *IL-18*), and (**D**–**F**) TJ proteins (occludin [*OCLN*], zonula occludens-1 [*ZO1*], claudin [*CLDN1*]) quantified by RT-qPCR. Spearman’s correlation. Two tails’ significance level adjusted at 0.05, *n* = 19.

First, we determined the potential relationship between CHGA and epithelial barrier functional markers. Based on our previous data [15], in samples pooled from patients with active UC and healthy individuals, CHGA mRNA expression demonstrated a strong significant positive correlation with *COL1A2* (*r* > 0.7, *p* < 0.0001) (Figure 1A) and epithelial-associated cytokines *IL-8* (*r* > 0.7, *p* < 0.0003), *IL-18* (*r* > −0.8, *p* < 0.0001) (Figure 1B,C). Counter-wise, CHGA mRNA expression revealed a negative correlation with mRNA TJ proteins expression *OCLN* (*r* < −0.7, *p* < 0.0004), *ZO1* (*r* < −0.6, *p* < 0.003) and *CLDN1* (*r* < −0.5, *p* < 0.01) (Figure 1D,E).

### 2.2. Deletion of Chga Attenuates Collagen Expression and Deposition in Acute DSS-Induced Colitis

**Figure 2:**
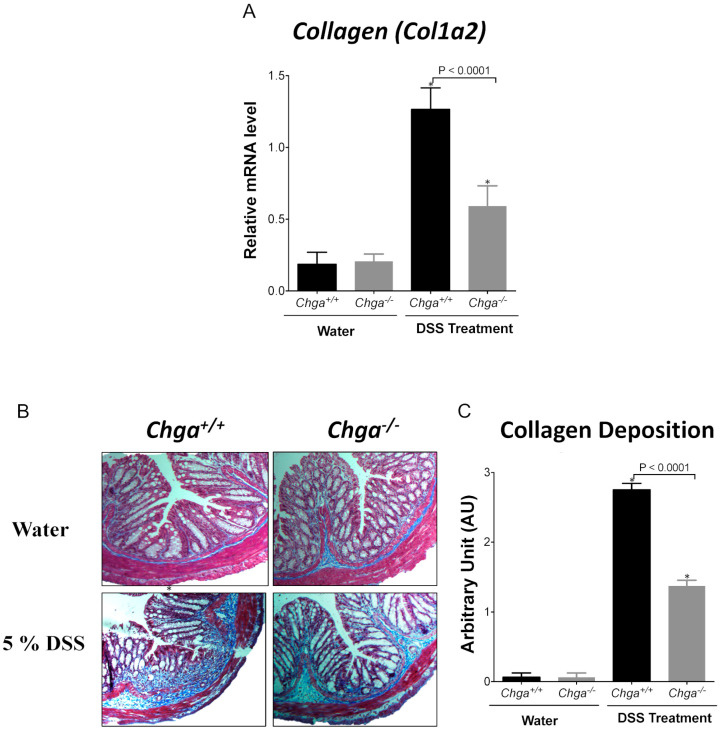
Deletion of chromogranin-A gene (*Chga*) attenuates collagen deposition and expression in acute dextran sulphate sodium (DSS)-induced colitis. Wild-type (*Chga^+/+^*) and *Chga*-knockout (*Chga^−/−^*) mice were given 5% DSS solution for five days in the drinking water to induce colitis. Control mice received water without DSS. Mice were sacrificed at day five, and colonic tissues were processed for further analysis. (**A**) RT-qPCR of *Col1a2* mRNA expression in colonic tissues. (**B**) Masson Trichrome staining (collagen stained in blue) at magnification of 100x, and (**C**) collagen deposition scores. One-way ANOVA followed by Tukey’s multiple comparison test. Each value represents the mean ± SEM, *n* = 6–8 per group. * *p* < 0.005 refers to significance compared with water treatment groups.

We recently reported that the deletion of *Chga* protects against DSS-induced colitis [15]. Herein, we demonstrated the impact of the lack of CHGA on the development of fibrosis. In WT mice, DSS administration significantly increased mRNA *Col1a2* colonic expression (Figure 2A), and the deletion of *Chga* significantly (*p* < 0.0001) decreased it (Figure 2A). Histologic staining confirmed the data were in WT mice colitis induction significantly increased the collagen deposition and its score (Figure 2B,C). Conversely, in *Chga^−/−^* mice, the lack of CHGA significantly (*p* < 0.0001) decreased the collagen deposition and its score (Figure 2B,C). In the absence of colitis, the lack of CHGA did not show any significant effects on mRNA *Col1a2* colonic expression and collagen deposition (Figure 2).

### 2.3. Deletion of Chga Maintains TJ Protein mRNA Expression and Decreases Colonic IL-18 Expression and Release in Acute DSS-Induced Colitis

**Figure 3:**
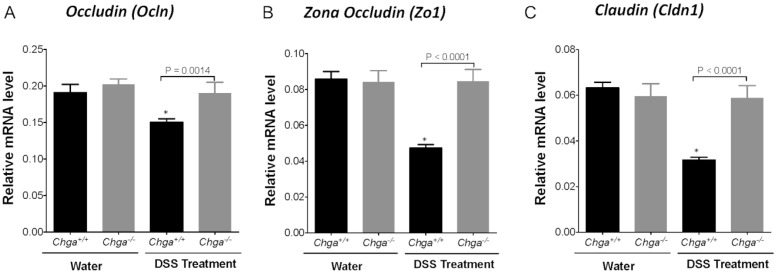
Deletion of chromogranin-A gene (*Chga*) maintains colonic tight junction (TJ) mRNA expression proteins in acute dextran sulphate sodium (DSS)-induced colitis. Wild-type (*Chga^+/+^*) and *Chga*-knockout (*Chga^−/−^*) mice were given 5% DSS solution for five days in the drinking water to induce colitis. Control mice received water without DSS. Mice were sacrificed at day five, and colonic tissues were processed for mRNA expression. (**A**–**C**) Whole colonic mRNA expression of tight junction (TJ) proteins (occludin [*Ocln*], zonula occludens-1 [*Zo1*], claudin [*Cldn1*]) quantified RT-qPCR. One-way ANOVA followed by Tukey’s multiple comparison test. Each value represents the mean ± SEM, *n* = 6–8 per group. * *p* < 0.005 refers to significance compared with water treatment groups.

**Figure 4:**
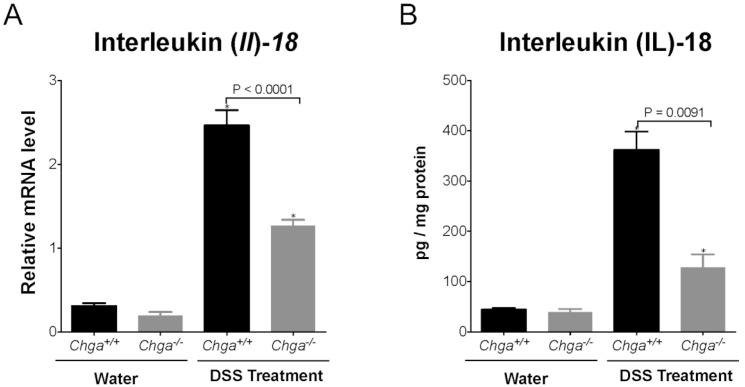
Deletion of chromogranin-A gene (*Chga*) reduces interleukin (IL)-8 & IL-18 mRNA expression and release in acute dextran sulphate sodium (DSS)-induced colitis. Wild-type (*Chga^+/+^*) and *Chga*-knockout (*Chga^−/−^*) mice were given 5% DSS solution for five days in the drinking water to induce colitis. Control mice received water without DSS. Mice were sacrificed at day five, and whole colonic tissues were processed for mRNA expression and ELISA. (**A**) Colonic *Il-18* mRNA expression quantified by RT-qPCR; (**B**) Colonic protein level of IL-18 quantified by ELISA. One-way ANOVA followed by Tukey’s multiple comparison test. Each value represents the mean ± SEM, *n* = 6–8 per group. * *p* < 0.005 refers to significance compared with water treatment groups.

Compared with non-colitic mice, colitic *Chga*-WT mice whole colonic sections exhibited a significant decrease in TJ proteins mRNA expression (*Ocln*, *Zo1*, *Cldn1*) (Figure 3A–C). The deletion of *Chga* abrogated this deleterious effect by maintaining TJ proteins mRNA expression (Figure 3). In parallel, colitic *Chga*-WT mice whole colonic sections demonstrated a significant upregulation in IL-18 mRNA expression (Figure 4A) and protein level (Figure 4B). However, in the absence of *Chga*, IL-18 mRNA and protein expression and IL-18 levels were significantly (*p* < 0.009 and 0.0001 respectively) decreased when compared with *Chga*-WT mice (Figure 4). In the absence of colitis, the lack of CHGA did not show any significant effects on IL-18 mRNA expression and protein level and TJ proteins mRNA expression (Figure 3 and Figure 4).

### 2.4. Chga-Deficient M2-Conditioned Supernatant Maintains TJ Protein mRNA Expression and Decreases IL-8 and IL-18 Expression and Release in DSS-Stimulated Human Caco-2 Epithelial Cells

**Figure 5:**
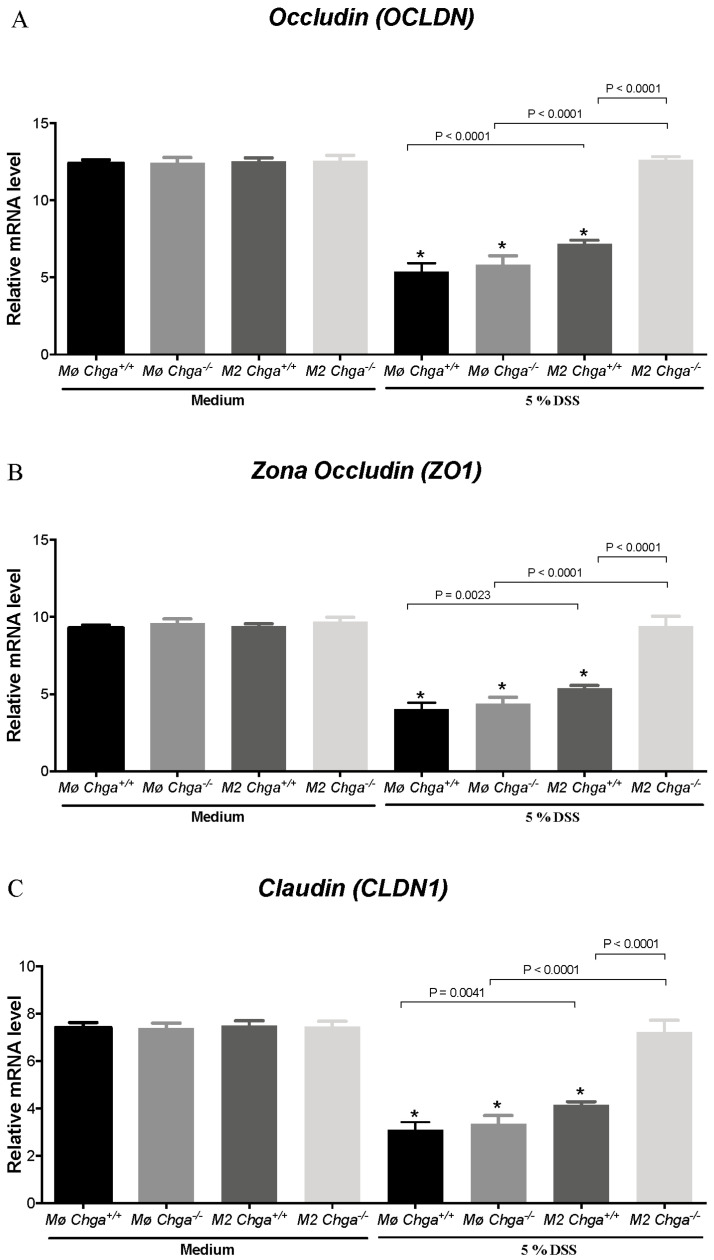
Chromogranin-A (*Chga*)-deficient alternatively activated macrophages (M2)-conditioned supernatant maintains the levels of tight junction (TJ) proteins in dextran sulphate sodium (DSS)-stimulated Caco-2 epithelial cells. Caco-2 cells were seeded at 3 × 10^5^ cells/well onto tissue culture plates and treated with 2 mL of non-conditioned supernatant (Mo) or M2-conditioned supernatant of wild-type (*Chga^+/+^*) and *Chga*-knockout (*Chga^−/−^*) for 24 h. Then cells were challenged with 5% DSS or regular medium for an additional 24 h. (**A**–**C**) Tight junction (TJ) proteins (occludin [*OCLN*], zonula occludens-1 [*ZO1*], claudin [*CLDN1*]) mRNA expression quantified by RT-qPCR in DSS-stimulated epithelial cells. One-way ANOVA followed by Tukey’s multiple comparison tests. Each value represents the mean ± SEM *n* = 6–9 per group. * *p* < 0.05 refers to significance compared with medium groups. Each experiment was repeated at least three times.

**Figure 6:**
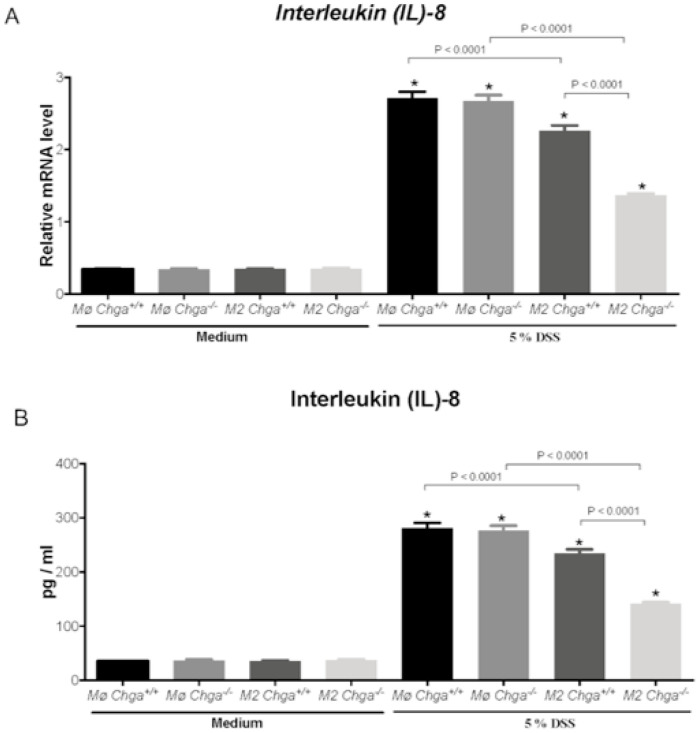
Chromogranin-A (*Chga*)-deficient alternatively activated macrophages (M2)-conditioned supernatant reduces interleukin (IL)-8 mRNA expression and release in dextran sulphate sodium (DSS)-stimulated Caco-2 epithelial cells. Caco-2 cells were seeded at 3 × 10^5^ cells/well onto tissue culture plates and treated with 2 mL of non-conditioned supernatant (Mo) or M2-conditioned supernatant of wild-type (*Chga^+/+^*) and *Chga*-knockout (*Chga^−/−^*) for 24 h. Then cells were challenged with 5% DSS or regular medium for an additional 24 h. (**A**) *IL-8* mRNA expression were quantified by RT-qPCR; (**B**) IL-8 releases were quantified by ELISA. One-way ANOVA followed by Tukey’s multiple comparison tests. Each value represents the mean ± SEM, *n* = 6–9. * *p* < 0.05 refers to significance compared with medium groups.

**Figure 7:**
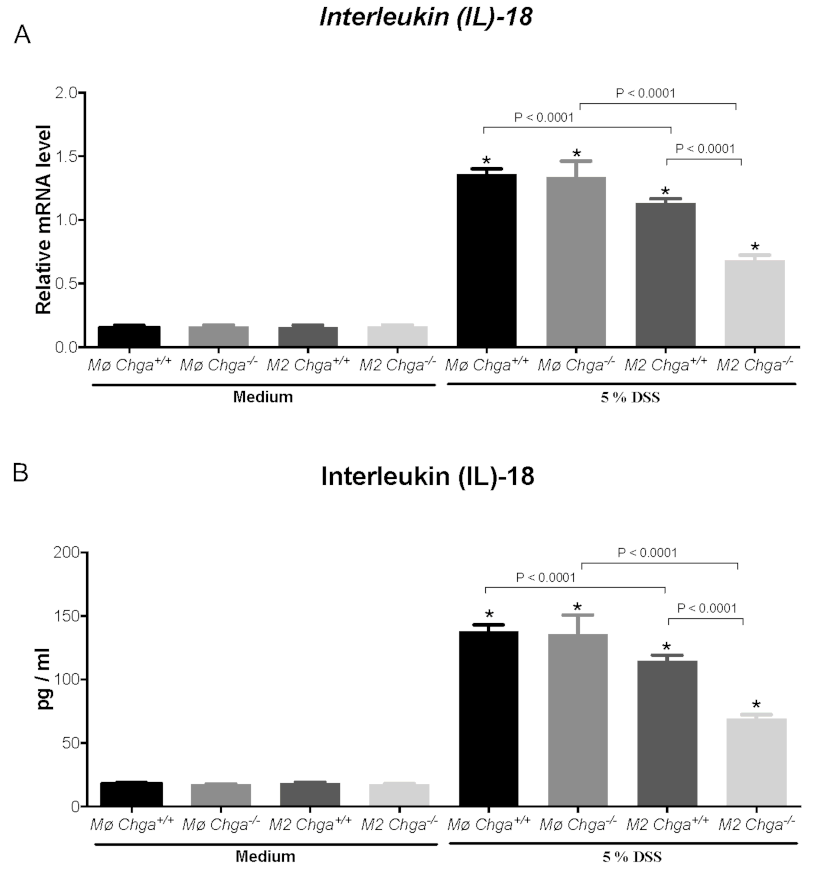
Chromogranin-A (*Chga*)-deficient alternatively activated macrophages (M2)-conditioned supernatant reduces interleukin (IL)-18 mRNA expression and release in dextran sulphate sodium (DSS)-stimulated Caco-2 epithelial cells. Caco-2 cells were seeded at 3 × 10^5^ cells/well onto tissue culture plates and treated with 2 mL of non-conditioned supernatant (Mo) or M2-conditioned supernatant of wild-type (*Chga^+/+^*) and *Chga*-knockout (*Chga^−/−^*) for 24 h. Then cells were challenged with 5% DSS or regular medium for an additional 24 h. (**A**) *IL-18* mRNA expression were quantified by RT-qPCR; (**B**) IL-18 releases were quantified by ELISA. One-way ANOVA followed by Tukey’s multiple comparison tests. Each value represents the mean ± SEM, *n* = 6–9. * *p* < 0.05 refers to significance compared with medium groups.

As previously described, the lack of *Chga* enhanced M2 polarization and the release of anti-inflammatory markers of M2 polarized peritoneal macrophages [15]. Therefore, to gain mechanistic insight, we compared the interaction between in vitro M2 polarized peritoneal macrophages obtained from naïve *Chga^+/+^* and *Chga^−/−^* mice and human IECs. Our previously reported data [19] demonstrated that the exposition of Caco-2 cell to 5% DSS induced a significant downregulation of TJ mRNA expression (*OCLN*, *ZO1*, *CLDN1)*. Here, we demonstrated the same effect when in the presence of WT or *Chga^−/−^* non-conditioned supernatant (Figure 5A–C). The presence of WT or *Chga^−/−^* M2-conditioned supernatant significantly increased TJ proteins mRNA expression when compared with non-conditioned supernatant (Figure 5). However, *Chga^−/−^* M2-conditioned supernatant reverted totally the epithelial homeostasis by maintaining TJ proteins mRNA and a significant (*p* < 0.0001) difference was observed between WT and *Chga^−/−^* M2-conditioned supernatants (Figure 5). In parallel, in colitic conditions, the presence of WT or *Chga^−/−^* non-conditioned supernatant significantly increased IL-8 and IL-18 mRNA expression (*p* < 0.0001) and release (*p* < 0.0001) (Figure 6 and Figure 7). WT or *Chga^−/−^* M2-conditioned supernatants significantly decreased IL-8 and IL-18 mRNA expression and release when compared with non-conditioned supernatant. Interestingly, a significant (*p* < 0.0001) difference was seen between WT and *Chga^−/−^* M2-conditioned supernatants. In the absence of DSS, WT or *Chga^−/−^* non- or M2-conditioned supernatant treatments did not show any significant effects on TJ protein and IL-8/18 mRNA expression and release (Figure 6 and Figure 7).

### 2.5. Chga-Deficient M2-Conditioned Supernatant Enhances the Functional Abilities of Human Caco-2 Epithelial Cells

**Figure 8:**
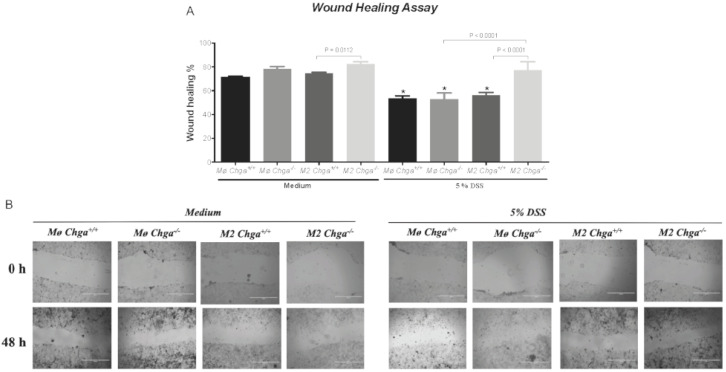
Chromogranin-A (*Chga*)-deficient alternatively activated macrophages (M2)-conditioned supernatant maintains the functional properties of dextran sulphate sodium (DSS)-stimulated Caco-2 epithelial cells. Caco-2 cells were seeded at 3 × 10^5^ cells/well onto tissue culture plates and treated with 2 mL of non-conditioned supernatant (Mo) or M2-conditioned supernatant of wild-type (*Chga^+/+^*) and *Chga*-knockout (*Chga^−/−^*) for 24 h. Then cells were challenged with 5% DSS or regular medium for an additional 24 h. (**A**, **B**) Epithelial cell migration (wound healing assay) at magnification of 100x. One-way ANOVA followed by Tukey’s multiple comparison tests. Each value represents the mean ± SEM, *n* = 6. * *p* < 0.05 refers to significance compared with medium groups. Each experiment was repeated at least three times.

**Figure 9:**
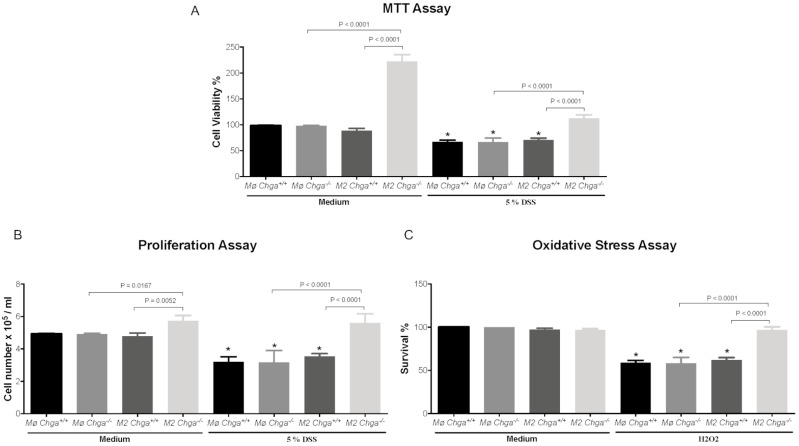
Chromogranin-A (*Chga*)-deficient alternatively activated macrophages (M2)-conditioned supernatant maintains the functional properties of dextran sulphate sodium (DSS)-stimulated Caco-2 epithelial cells. Caco-2 cells were seeded at 3 × 10^5^ cells/well onto tissue culture plates and treated with 2 mL of non-conditioned supernatant (Mo) or M2-conditioned supernatant of wild-type (*Chga^+/+^*) and *Chga*-knockout (*Chga^−/−^*) for 24 h. Then cells were challenged with 5% DSS or regular medium for an additional 24 h. (**A**) intestinal epithelial cell viability (assessed by the 3-[4–dimethyl thiazolyl-2yl]-2, 5-diphenyl tetrazolium [MTT]), (**B**) proliferation, and (**C**) epithelial cells oxidative stress assay. One-way ANOVA followed by Tukey’s multiple comparison tests. Each value represents the mean ± SEM, *n* = 6. * *p* < 0.05 refers to significance compared with medium groups. Each experiment was repeated at least three times.

Next, we investigated the potential effects of *Chga^−/−^* M2-conditioned supernatant on colonic human Caco-2 cells’ functions in an unstimulated and DSS-stimulated environment. In the presence of WT or *Chga^−/−^* non-conditioned supernatant, DSS-stimulated cells demonstrated a significant decrease in wound healing, viability and proliferation assays (Figure 8 and Figure 9), and the presence of WT M2-conditioned supernatant did not alter the effects. However, *Chga^−/−^* M2-conditioned supernatant abrogated these deleterious effects (Figure 8 and Figure 9) and a significant (*p* < 0.0001) difference was observed between WT and *Chga^−/−^* M2-conditioned supernatants. Moreover, in the presence of WT or *Chga^−/−^* non-conditioned supernatant, the survival rate of Caco-2 cells treated with H_2_O_2_ was significantly decreased compared with untreated cells, (Figure 9C), and the presence of WT M2-conditioned supernatant did not alter the effect. However, *Chga^−/−^* M2-conditioned supernatant abrogated these deleterious effects (Figure 9C) and a significant (*p* < 0.0001) difference was observed between WT and *Chga^−/−^* M2-conditioned supernatants. In the absence of DSS, WT or *Chga^−/−^* non-conditioned supernatant treatments did not show any significant effects on all the assay performed (Figure 8 and Figure 9). Interestingly, *Chga^−/−^* non-conditioned supernatant treatments increased significantly the wound healing, MTT (3-(4,5-dimethylthiazol-2-yl)-2,5-diphenyltetrazolium bromide) and proliferation assay (Figure 9A,B).

## 3. Discussion

We recently reported that CHGA & its derived peptides are involved in IBD’s pathophysiology through various extracellular and intracellular mechanisms [2,6,15,16,17,18,19,20]. For the first time, the present study showed the interdependence between CHGA, TJ proteins and the epithelial functions in humans and rodents using in-vivo and in-vitro models. We demonstrated that in human mucosal clinical biopsies, the expression of CHGA has a strong linear negative correlation with TJ proteins and a significant linear positive correlation with the epithelial cells associated cytokines (IL-8, IL-18) and collagen. Moreover, in a rodent model, the deletion of *Chga* ameliorates DSS-induced colitis by reducing the colonic expression of collagen, IL-18, by maintaining the epithelial barrier’s function and preserving the decrease of TJ proteins’ expression. Furthermore, *Chga-*deficient M2-conditioned supernatant maintained TJ proteins’ levels, reduced epithelial cells-associated cytokines (IL-8, IL-18) release, and held the epithelial cells’ functional capacities of DSS-stimulated Caco-2 epithelial cells. Taken together, our data demonstrate that CHGA is an important player in the innate immune and epithelial cells responses in the intestinal mucosa during the development of inflammation.

Immune dysregulation, disruption of epithelial barrier’s functions and high levels of CHGA are some of the hallmarks of IBD [2,15,21]. Moreover, severe intestinal injury and inflammation can induce excessive transmural extracellular matrix collagen deposition accompanied by an alteration of tissue architecture [22]. The correlations depicted between CHGA and TJ proteins, collagen and epithelial cells associated cytokines in the human subjects suggests a pro-inflammatory role of CHGA. Several studies support our data as they reported that CHGA demonstrates a positive correlation with the clinical severity of intestinal inflammation and responsiveness to biologic therapy in IBD patients [13,14,15,23,24,25]. In IBD, chemokine ligand 8 (known as IL-8) is secreted by IECs and serves as a chemoattractant for polymorphonuclear leukocytes [19]. Recently in the context of colitis, a close relation between IL-8 and CHGA-derived peptide, vasostatin-1 has been reported, where the severity of experimental colitis was decreased through inhibition of human IECs IL-8 production [26]. Moreover, it was demonstrated that CHGA-derived peptide, chromofungin, correlated positively with M2 and gene expression of TJ proteins and negatively with IL-8, IL-18 and collagen gene expression in humans [6]. Furthermore, the negative relationship between CHGA and M2-associated mediators (tumor growth factor beta, arginase 1 or nitric oxide) [15] in patients with UC favours our findings. Taken together, it can be suspected that CHGA plays directly or indirectly a significant role in regulating the inflammatory cascades.

Over the last ten years, it has been demonstrated that the development of experimental UC-like colitis increases intestinal permeability, loss of TJ proteins [27,28] is associated with an upregulation of pro-inflammatory cytokines (IL-18) [6,17]. It is suspected that the two first events can predate the development of an inflammatory response [29,30]. To explore the relationship between CHGA and those markers, we used *Chga^−/−^* mice and a DSS model of colitis. Interestingly, we found that the deletion of *Chga* reduced the collagen deposition, maintained TJ proteins and decreased the expression and the release of IL-18 in the inflamed colon. This suggests that the deletion of CHGA plays a protective role, resulting in decreasing the inflammatory processes and protecting tissue from destruction. This is consistent with our previous study demonstrating attenuation of DSS-induced acute colitis in *Chga^−/−^* mice via decreasing programmed cell death [15]. IL-18 has been demonstrated to be an important regulatory protein in the homeostatic and pathophysiological context as its decrease is associated with reduction in the severity of DSS-induced colitis [6,17]. Moreover, its absence can sustain epithelial maintenance but also decrease the development of colitis [19]. In our study *Chga^−/−^* colitic mice saw an increase in microscopic collagen deposition and quantification, which can be elicited by IL-18 [19]. We did not quantify IL8, as in mice, the homolog of human IL-8 is absent from their genome. Overall, our experimental plan is in line with others, demonstrating the possible impact of peptides on maintaining TJ proteins, decreasing collagen deposition and IL-18 [17,19]. Here, we provide additional evidence on the potential underlying pathways and relation between the endocrine and immune system, seen during the improvement of DSS- induced colitis in the absence of *Chga* [15].

UC is characterized by epithelial erosions, ulcers, and decreased epithelial repair mechanisms [31]. Macrophage activation and infiltration are two of the hallmarks of the activation of the innate immune response. The unbalance between M1 and M2 can induce a defect of intestinal epithelial repairs in IBD [32,33]. M2 mediate protective effects via various mechanisms, including maintenance or the rebuilding of the epithelial barrier through cell proliferation and migration, and promoting resistance to epithelial apoptosis induced by oxidative stress [33]. As previously described, the lack of *Chga* enhanced M2 polarization and the release of anti-inflammatory markers [15]. Therefore, to gain mechanistic insight, we compared the interaction between in vitro M2 polarized peritoneal macrophages supernatant obtained from naïve *Chga^+/+^* and *Chga^−/−^* mice and human IECs. According to the aforementioned data, *Chga^−/−^* M2-conditioned supernatant increased cell migration, proliferation, viability, and the survival of caco-2 epithelial cells. Furthermore, our study revealed that the beneficial effects of the lack of *Chga* might contribute to the epithelium’s resistance to the harmful effects of oxidative stress. It is not clear why, in control conditions *Chga^−/−^* M2-conditioned supernatant, the wound healing, viability and the proliferation assay were significantly increased when compared with WT non-conditioned supernatant. It is possible in a non-inflammatory state that the qualitative modification of phenotype seen on M2 are enough in to engage a modification of these mechanisms, compared to other where there is a need in a deleterious trigger. Oxidative stress and subsequent epithelial apoptosis are being critical features of inflammation and have been shown in colitis development [34,35]. In our experiment, *Chga^−/−^* M2-conditioned supernatant protected Caco-2 cells from cell death elicited by the free radical donor, H2O2, emphasizing the importance of the CHGA in regulating physiological functions well as pathological conditions as demonstrated by others using other components [15,17,19].

Several limitations to our studies exist. First, the correlation analysis we conducted was based on all the samples pooled together. Therefore, it is possible that our control sample could have a lower score compared to active UC samples. Ultimately a linear analysis should have been assessed for correlation within each group. Due to the limitation of samples, we were not able to conduct an independent analysis. Second, in this set of experiments, we assessed the mRNA level based on the concept linking DNA, RNA and proteins and suggesting a direct relationship between mRNA and protein levels [17]. However, in some studies and some specific conditions, the mRNA levels do not correlate with protein expression levels or protein function [19]. Therefore, it is conceivable that other regulatory mechanisms exist. Third, it would be essential to localize proteins’ expression and assess ex-vivo permeability using a USSING chamber technique.

It is possible that other factors contributed to the beneficial effect on the intestinal inflammation in this study, especially the potential impact on the gut microbiome in our in-vivo model. Gut microbiota plays an essential role in the development of IBD [2,16]. We have shown that *Chga*-deficient mice developed a specific type of microbiota from their littermate WT mice. Moreover, studies have demonstrated the impact of several CHGA-derived peptides on the gut microbiota [2,16]. Furthermore, the regulation of epithelial proliferation and differentiation requires modulation of the metabolic energy through AMP-Activated Protein Kinase (AMPK). AMPK is a master regulator of the cellular energy and the Caudal type homeobox 2 (CDX2) known as an essential transcription factor governing differentiation of intestinal epithelial cells [36]. Therefore, further studies are required to investigate the potential interplay between CHGA/gut microbiota, colonic permeability, and AMPK/CDX2. In humans and in our in-vivo model, other innate and adaptive immune components can also play a major, including antigen-presenting cells (e.g., dendritic cells) or T or B cells, all well known to play a role in colonic inflammation development [37,38].

In conclusion, herein, we described a protective effect of *Chga^−/−^* M2-conditioned supernatant on TJ proteins and epithelial cells’ functions in an inflamed environment. The purpose of this descriptive study is to open a new field of research and to stimulate other groups of researchers working in the same field of research. These findings provide additional clinical relevance to how CHGA could play an important role in mucosal protection in UC patients. In the long-term, as UC is characterized by epithelial erosions, ulcers, and decreased epithelial barrier integrity [31], specific treatment targeting CHGA or its derived peptides might be of interest to improve epithelial proliferation and migration.

## 4. Material and Methods

### 4.1. Active Ulcerative Colitis & Control Subjects

This study was approved by the University of Manitoba Health Research Ethics Board (Project ID: HS14878 [E], Approval date: January 8, 2018). Persons undergoing colonoscopy with either known IBD or without IBD gave consent for the collection of biopsies. Four biopsies were taken from inflamed sites that were adjacent to tissues harvested for histopathologic assessment in subjects with active UC (*n* = 10) and inactive (mild) UC (*n* = 9). Biopsies were taken from comparable, un-inflamed sites in healthy subjects (*n* = 10). For additional clinical details, see Kermarrec et al. [39]. Biopsies were used immediately for further RNA extraction and gene expression analyses.

### 4.2. Chga-Knockout Mice

Experiments were approved by the University of Manitoba Animal Ethics Committee (Project ID: 15-010, Approval date: January 25, 2018) and conducted under the Canadian guidelines for animal research. Mice heterozygous for *Chga* expression (*Chga^+/−^*) [40] on a C57BL/6 background were used to generate *Chga^+/+^* and *Chga^−/−^.*

### 4.3. DSS Injury Model

**Figure 10:**
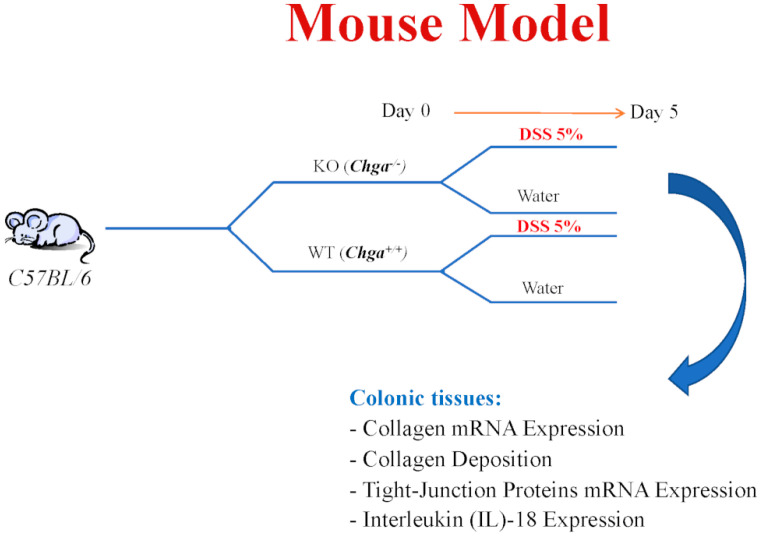
Mouse model: Experimental design.

DSS (molecular weight [MW], 40 kDa: MP Biomedicals, Soho, OH) was added to the drinking water at a final concentration of 5% (wt/vol) for 5 days [41,42] to 6–8-week-old mice (Figure 10). Controls were time-matched and consisted of mice that received regular drinking water only. Mean DSS consumption was noted per cage each day.

### 4.4. Assessment of Colitis Severity and the Collagen Deposition

Collagen deposition and fibrosis scores were assessed. Formalin (Sigma, Mississauga, ON, Canada)-fixed colon segments coming from the splenic flexure were paraffin (Sigma, Mississauga, ON, Canada)-embedded and 3 mm sections were stained using Masson’s Trichrome to quantify collagen deposition (Sigma, Mississauga, ON, Canada) [19].

### 4.5. Protein Assay

Colonic samples were homogenized mechanically in 700 μL of Tris HCl buffer containing protease inhibitors (Sigma, Mississauga, Canada), then centrifuged for 30 min, and supernatants were frozen at 80 °C until assay [20]. Commercial enzyme-linked immunosorbent assays (ELISAs) kits for mouse and/or human IL-18 and IL-8 (R&D Systems, Inc., Minneapolis, MN, USA) were used according to the manufacturer’s instructions.

### 4.6. Intraperitoneal Macrophages Cell Culture

Peritoneal macrophages were isolated from naïve Chga^−/−^ and Chga^+/+^ mice, as described by Mosser and Zhang [43]. Isolated macrophages were cultured in Dulbecco’s Modified Eagle Medium (DMEM) with 10% fetal bovine serum (FBS) for 48 h with daily medium changes. Cells were then serum-starved overnight in DMEM with low FBS (0.5%) and then exposed for 6 h to vehicle or interleukin (IL)-4/IL-13 (20 ng/mL) (R&D Systems, Inc., Minneapolis, MN, USA) to promote an M2 profile. Supernatants from non-or M2-conditioned were harvested for further analysis.

### 4.7. Cell Line Culture 

**Figure 11:**
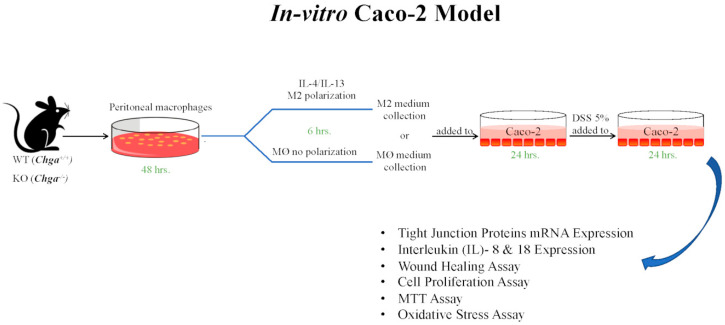
Experimental design: Caco-2 culture.

The interactions between human IECs and supernatants from non-conditioned and M2-conditioned obtained from naïve *Chga^+/+^* and *Chga^−/−^* mice were studied. Human intestinal epithelial cell line, Caco-2 (ATCC, Manassas, VA, USA), was cultured and handled as described previously [19] in Eagle’s Minimum Essential Medium (EMEM) (glutamine, high glucose) supplemented with 100 U/mL penicillin, 100 μg/mL streptomycin, and 20% deactivated FBS was used. The cell culture medium was changed every three days until the cells were fully differentiated (80–90% confluent). For each experimental setup, three separate experiments were performed, and at least six wells per condition were assigned. Caco-2 cells were seeded at 3 × 10^5^ cells/well onto tissue culture plates and treated with 2 mL of non- or M2-conditioned supernatant for 24 h. Then cells were challenged with 5% DSS or regular medium for an additional 24 h (Figure 11). Gene expression of TJ proteins (*OCLN*, *ZO1*, *CLDN1*), pro-inflammatory cytokines IL-8 and IL-18, migration, proliferation, viability, using 3-(4,5-Dimethylthiazol-2-yl)-2,5-diphenyltetrazolium bromide for MTT assay (Trevigen Inc., Gaithersburg, MD, USA) according to the manufacturer’s instructions. The oxidative stress assay 2 mL of 200 mmol/L of H2O2 in PBS were given to the Caco-2 cells for 30 min then Trypan blue staining was performed to count viable cells to evaluate survivability of Caco-2 cell line were evaluated as we described before [18].

### 4.8. Gene Expression 

RNA from tissues or cells were extracted using TRIzol (Gibco BRL, Life Technologies, Grand Island, NY, USA) and reverse-transcribed using SuperScript VILO cDNA Synthesis Master Mix (Invitrogen, Grand Island, NY, USA). Gene expression was measured with a quantitative polymerase chain reaction in a Roch Light Cycler 96 Real-Time System using Power SYBR green master mix (Life Technologies, Burlington, ON, Canada). Differences in the threshold cycle (Δ*C*_t_) number between the target genes and the optimal reference genes [44,45] were used to calculate differences in expression. Primer sequences are provided in Table 1 and Table 2.

### 4.9. Data Analysis

Data are expressed as the mean ± standard error of the mean (SEM). Statistical analyses were performed using unpaired a Mann-Whitney U test, and one—and two-way ANOVAs followed by a post-hoc Tukey test when appropriate. Spearman’s correlation test was used. Statistical significance was concluded at *p* < 0.05. Statistics were computed using GraphPad Prism software (version 6; GraphPad Software, Inc, La Jolla, CA, USA).

## Figures and Tables

**Table 1:** Human primers sequences.

Gene Name	Forward	Reverse
***CLDN1***	AGGTGCTATCTGTTCAGTGATG	TGGCTGACTTTCCTTGTGTAG
***COL1A2***	GAGCGGTAACAAGGGTGAGC	CTTCCCCATTAGGGCCTCTC
***IL18***	GCGTCACTACACTCAGCTAAT	GCGTCACTACACTCAGCTAAT
***IL8***	ACTGAGAGTGATTGAGAGTGGAC	AACCCTCTGCACCCAGTTTTC
***OCLDN***	ACAAGCGGTTTTATCCAGAGTC	GTCATCCACAGGCGAAGTTAAT
***TBP***	CCCGAAACGCCGAATATAATCC	AATCAGTGCCGTGGTTCGTG
***ZO1***	CCAGCCTGCTAAACCTACTAAA	ATCTCTTGCTGCCAAACTATCT

**Table 2:** Mouse primers sequences.

Gene	Forward	Reverse
***Cldn1***	GGGGACAACATCGTGACCG	AGGAGTCGAAGACTTTGCACT
***Col1a2***	GGTGAGCCTGGTCAAACGG	ACTGTGTCCTTTCACGCCTTT
***Eef2***	TGTCAGTCATCGCCCATGTG	CATCCTTGCGAGTGTCAGTGA
***Il18***	GACTCTTGCGTCAACTTCAAGG	CAGGCTGTCTTTTGTCAACGA
***Ocldn***	TTGAAAGTCCACCTCCTTACAGA	CCGGATAAAAAGAGTACGCTGG
***Zo1***	GCCGCTAAGAGCACAGCAA	TCCCCACTCTGAAAATGAGGA
